# Clinical efficacy of SGLT2 inhibitors with different SGLT1/SGLT2 selectivity in cardiovascular outcomes among patients with and without heart failure: A systematic review and meta-analysis of randomized trials

**DOI:** 10.1097/MD.0000000000032489

**Published:** 2022-12-23

**Authors:** Mei-Chuan Lee, Yi-Ming Hua, Chun-Ting Yang, Fang-Hsiu Kuo, Wei-Ting Chang, Hsin-Ju Tang, Han Siong Toh, Yu-Min Lin, Sih-Yao Chen, Hung-Yu Chang, Chia-Te Liao

**Affiliations:** a Department of Pharmacy, Chi Mei Medical Center, Tainan, Taiwan; b Department of Public Health, College of Medicine, National Cheng Kung University, Tainan, Taiwan; c Department of Pharmacy, College of Medicine, National Cheng Kung University, Tainan, Taiwan; d Division of Cardiology, Department of Internal Medicine, Chi Mei Medical Center, Tainan, Taiwan; e Institute of Clinical Medicine, College of Medicine, National Cheng Kung University, Tainan, Taiwan; f Department of Biotechnology, Southern Taiwan University of Science and Technology, Tainan, Taiwan; g Chang Gung University of Science and Technology, Chiayi, Taiwan; h Department of Intensive Care Medicine, Chi Mei Medical Center, Tainan, Taiwan; i Department of Health and Nutrition, Chia Nan University of Pharmacy & Science, Tainan, Taiwan; j Faculty of Medicine, School of Medicine, National Yang Ming Chiao Tung University, Taipei, Taiwan; k Heart Center, Cheng Hsin General Hospital, Taipei, Taiwan.

**Keywords:** heart failure (HF), hospitalization for HF (HHF), mortality, SGLT2/SGLT1 selectivity, sodium-glucose co-transporter-2 (SGLT2) inhibitor

## Abstract

Some sodium-glucose co-transporter-2 (SGLT2) inhibitors showed benefits on heart failure (HF), but different SGLT2/SGLT1 selectivity might influence the treatment effect. This study aimed to meta-analyze the treatment effects of SGLT2 inhibitors and the diversity of receptor selectivity for patients with and without HF.

**Methods:**

Randomized controlled trials were searched in PubMed, Embase, Cochrane databases and ClinicalTrials.gov registry from inception to October 2020. The interest outcomes were analyzed with random-effects models and presented with a risk ratio (RR) and 95% confidence interval (CI). Subgroup analyses examined the treatment effects among SGLT2 inhibitors with different SGLT2/SGLT1 selectivity.

**Results:**

The final analyses included 10 trials and 52,607 patients. The RR of total cardiovascular (CV) death or hospitalization for HF (HHF) between SGLT2 inhibitors and placebo was 0.79 (95% CI 0.74–0.84, *I*^2^ = 31%). With SGLT2 inhibitors, HF patients had reduced mortality risks (RR 0.89, 95% CI 0.80–0.99, *I*^2^ = 0), and non-HF patients had lower risks of major adverse CV events (RR 0.92, 95% CI 0.85–0.99, *I*^2^ = 0). The risk reduction of HHF was consistent in groups of HF (RR 0.72, 95% CI 0.64–0.80, *I*^2^ = 8%) and non-HF (RR 0.74, 95% CI 0.61–0.89, *I*^2^ = 0), but the effect of the low SGLT2/SGLT1 selectivity inhibitor was insignificant in non-HF patients.

**Conclusion:**

The efficacy of SGLT2 inhibitors on risk reduction of total CV death or HHF is consistent with the previous studies. The regimen is beneficial for reducing mortality in patients with HF and major adverse CV events in those without HF. Different SGLT2/SGLT1 selectivity may differ in the treatment effects in patients with and without HF.

## 1. Introduction

Heart failure (HF) is a clinical syndrome resulting from most cardiovascular (CV) diseases, and the symptoms occur as the cardiac output cannot provide proper perfusion to support the body’s needs.^[1]^ Due to the aging population, the HF prevalence is increasing, estimated at 1% to 2% of the global population.^[2]^ Besides, HF causes high mortality and morbidity, and the patient’s quality of life are usually poor. This situation has burdened healthcare systems worldwide over the decades despite the advances in treatment and prevention.

Current HF therapies are based on clinical trials particularly designed for patients with HF and reduced ejection fraction, and the primary goals are the reduction of mortality and hospitalization for HF (HHF). The pharmacological strategies rely on neurohormonal blockades, that is, renin–angiotensin–aldosterone system inhibitors and β-blockers.^[3,4]^ However, these medications are insufficient for the entire HF population, and factors such as the various patient’s underlying conditions and the individual drug adverse effects might restrain the treatment options, for example, worsened renal function and hypotension.

Sodium-glucose co-transporter 2 (SGLT2) inhibitors have been used to reduce blood sugar in type 2 diabetes by increasing urinary glucose excretion.^[5]^ Apart from diabetes,^[6–8]^ large-scale randomized controlled trials (RCTs) have shown their efficacy in HF treatment, including the DAPA-HF, EMPEROR-Reduced, and SOLOIST-WHF trials.^[9–11]^ Nevertheless, the heterogeneity among these studies may influence the interpretation of CV outcomes. Besides, SGLTs can be classified by their modulatory sites, with more SGLT2 in the kidney and SGLT1 predominating in the intestine.^[12]^ Various structures, selectivity and pharmacokinetics of individual inhibitors might thus lead to different efficacy and safety according to their modulatory sites.^[13]^ Despite some meta-analyses,^[14–22]^ the clinical performance of SGLT2 inhibitors influenced by the diversity of different SGLT2/SGLT1 selectivity is still not well elucidated. Therefore, we aimed to perform a systematic review and meta-analysis to evaluate the treatment effects of SGLT2 inhibitors and examine the diversity of the different SGLT2/SGLT1 selectivity in patients with and without HF.

## 2. Materials and methods

### 2.1. Inclusion criteria

The RCTs assessing the effect of SGLT2 inhibitors on mortality risks or HHF were included, and the subjects with and without HF were all enrolled. We identified the studies with explicit inclusion and exclusion definitions and excluded those meeting the conditions: animal studies, observational studies, only including a protocol, duplicated studies, lacking CV outcomes, and non-English articles.

### 2.2. Search strategy and study selection

We searched and identified the relevant studies from inception to October 2020 in PubMed, Embase and Cochrane databases and collected the unpublished studies from the ClinicalTrials.gov registry (http://clinicaltrials.gov/). The main search keywords were heart failure, SGLT2 inhibitors and each SGLT2 inhibitor, and the details were listed in supplementary material S1, Supplemental Digital Content, http://links.lww.com/MD/I235. All retrieved articles, including manuscripts, abstracts and citations, were reviewed, and the other studies were selected using the references and corresponding with subject experts.

### 2.3. Data extraction and methodological quality appraisal

Two reviewers (MCL and YMH) independently extracted the baseline data, including study design, trial characteristics, patient’s comorbidity, underlying medication, the regimen of SGLT2 inhibitors, and outcomes, including mortality or hospitalizations for HF. Given the disagreements, another author (CTL) was invited to resolve them by discussion.

The risk of bias method based on the Cochrane Collaboration was used to assess the methodological quality of individual studies independently.^[23]^ Several domains were assessed by 2 reviewers (MCL and YMH), including the randomization and allocation, blinding and controlled designs, follow-up duration, patient’s withdrawal information, intention-to-treat analysis and freedom from other biases.

The primary outcome was a total incidence rate of CV mortality or HHF. The secondary outcome was the composite of major adverse cardiovascular events (MACE), including CV mortality, ischemic stroke or acute myocardial infarction, and individual clinical events.

### 2.4. Statistical analyses

The current meta-analyses were based on the Preferred Reporting Items for Systematic Reviews and Meta-Analyses guidelines.^[24]^ We calculated standard deviations from the given limits or standard errors and presented the dichotomous outcomes with a risk ratio (RR) and a 95% confidence interval (CI). The pooled RR and weighted mean differences were estimated using the DerSimonian and Laird random effects model.^[25]^ The Review Manager version 5.4 (The Cochrane Collaboration, Oxford, UK) was used for data input and analyses.

The Cochrane *Q* tests and *I*^2^ statistics were used to examine the inconsistency and statistical heterogeneity of treatment effects across the different studies. A *P* value <.10 on Cochrane *Q* tests was considered statistical significance. *I*^2^ statistics can quantify the proportion of the total outcome variability across the studies, and the values <25%, between 25% and 75%, and >75% presented low, moderate and high statistical heterogeneity, respectively. Advanced sensitivity analyses would be conducted to examine the uncertainty in the results if the values were >50%. We also performed subgroup analyses to examine the individual effects of SGLT2 inhibitors with different SGLT2/SGLT1 selectivity on HHF and mortality in the HF and non-HF groups. SGLT2 inhibitors with high selectivity included empagliflozin, dapagliflozin and ertugliflozin, while those with low selectivity (dual SGLT2/1-inhibitor effects) included sotagliflozin and canagliflozin.^[26]^

### 2.5. Ethical statement

This meta-analysis study was exempt from ethics review because this study retrieved and synthesized data from published studies in which informed consent had already been obtained in the trials.

## 3. Results

### 3.1. Eligible studies in research results

The literature search flowchart based on the Preferred Reporting Items for Systematic Reviews and Meta-Analyses guidelines is presented in Figure S1, Supplementary Digital Content, http://links.lww.com/MD/I236. A total of 2152 studies were selected in the initial search. After removing 649 duplicates, we screened the title and abstract of the 1503 identified studies, of which 1264 articles were excluded due to non-RCTs or not matching the inclusion criteria. Subsequently, we removed 34 articles because of the lack of full-text and published data or withdrawal from ClinicalTrials.gov and excluded 195 after the full-text assessment. Eventually, 10 RCTs with a qualitative synthesis of complete data were chosen for the final meta-analyses.^[6–11,27–30]^

### 3.2. Characteristics of the studies and populations included

Ten identified RCTs included dapagliflozin (the DEFINE-HF, DECLARE-TIMI 58, and DAPA-HF trials),^[8,9,27]^ canagliflozin (the CANVAS PROGRAM trial),^[6]^ empagliflozin (the EMPA-RESPONSE-AHF, EMPA-REG OUTCOME, and EMPEROR-Reduced trials),^[7,10,28]^ ertugliflozin (the VERTIS-CV trial),^[29]^ and sotagliflozin (the SOLOIST-WHF trial and SCORED trial).^[11,30]^

The final meta-analyses included 52,607 patients. The mean age in the EMPA-RESPONSE-AHF was the highest (79 and 73 years in the empagliflozin and controlled groups), while the DEFINE-HF patients were the youngest (62 and 60 years in the dapagliflozin and controlled groups). The most extended follow-up period was 4.2 years in the DECLARE-TIMI 58 trial,^[8]^ and the shortest was 12 weeks in the EMPA-RESPONSE-AHF trial^[28]^ (Table 1 and Table S1, Supplementary Digital Content, http://links.lww.com/MD/I237).

**Table 1:** Baseline characteristics of included studies.

Study	Follow-up time	Age (mean)	Female (%)	DM (%)	MI (%)	HF (%)	NT-pro BNP (mean, pg/mL)	eGFR (mean, mL/min/1.73 m^2^)	SBP (mean, mm Hg)	ACEI/ARB (%)	ARNI (%)	MRA (%)
CANVAS PROGRAM (canagliflozin)	188.2 wk	C: 63.2 ± 8.3 P: 63.4 ± 8.2	C: 35.1 P: 36.7	C: 100 P: 100	C: 55.8 P: 57.2	C: 13.9 P: 15.1	NA	C: 76.7 ± 20.3 P: 76.2 ± 20.8	C: 136.4 ± 15.8 P: 136.9 ± 15.8	Any RAAS inhibitor* C: 80.2 P: 79.8		
DECLARE-TIMI 58 (dapagliflozin)	4.2 yr	D: 63.9 ± 6.8 P: 64.0 ± 6.8	D: 36.9 P: 27.9	D: 100 P: 100	D: 32.9 P: 33	D: 9.9 P: 10.2	NA	D: 85.4 ± 15.8 P: 85.1 ± 16.0	D: 135.1 ± 15.3 P: 134.8 ± 15.5	D: 81.3 P: 81.3	NA	NA
DEFINE-HF (dapagliflozin)	13 wk	D: 62.2 ± 11 P: 60.4 ± 12	D: 27.5 P: 25.8	D: 61.8 P: 64.4	NA	D: 100 P: 100	D: 1136 P: 1136	D: 66.9 ± 21.1 P: 71.2 ± 23.1	NA	D: 58 P: 60.6	D: 35.9 P: 28.8	D: 58 P: 63.3
DAPA-HF (dapagliflozin)	18.2 mo	D: 66.2 ± 11.0 P: 66.5 ± 10.8	D: 23.8 P: 23.0	D: 41.8 P: 41.8	NA	D: 100 P: 100	D: 1428 P: 1446	D: 66.0 ± 19.6 P: 65.5 ± 19.3	D: 122.0 ± 16.3 P: 121.6 ± 16.3	D: 84.5 P: 82.8	D: 10.5 P: 10.9	D: 71.5 P: 70.6
EMPA-REG OUTCOME (empagliflozin)	3.1 yr	E: 63.1 ± 8.6 P: 63.2 ± 8.8	E: 28.8 P: 28	E: 100 P: 100	E: 46.7 P: 46.4	E: 9.9 P: 10.5	NA	E: 74.2 ± 21.6 P: 73.8 ± 21.1	E: 135.3 ± 16.9 P: 135.8 ± 17.2	E: 81 P: 80.1	NA	E: 6.5 P: 5.8
EMPA-RESPONSE-AHF (empagliflozin)	12 wk	E: 79 P: 73	E: 40 P: 16	E: 38 P: 28	E: 30 P: 38	E: 100 P: 100	E: 4406 P: 6168	E: 55 ± 18 P: 55 ± 18	E: 127 ± 22 P: 121 ± 25	E: 45 P: 50	E: 5 P: 3	E: 48 P: 45
EMPEROR-Reduced (empagliflozin)	16 mo	E: 67.2 ± 10.8 P: 66.5 ± 11.2	E: 23.5 P: 24.4	E: 49.8 P: 49.8	NA	E: 100 P: 100	E: 1887 P: 1926	E: 61.8 ± 21.7 P: 62.2 ± 21.5	E: 122.6 ± 15.9 P: 121.4 ± 15.4	E: 70.5 P: 68.9	E: 18.3 P: 20.7	E: 70.1 P: 72.6
VERTIS-CV (ertugliflozin)	3.5 yr	Er: 64.4 ± 8.1 P: 64.4 ± 8.0	Er: 29.7 P: 30.7	Er: 100 P: 100	Er: 47.7 P: 48.4	Er: 23.4 P: 24.5	NA	Er: 76.1 ± 20.9 P: 75.7 ± 20.8	Er: 133.5 ± 13.7 P: 133.1 ± 13.9	Er: 80.9 P: 81.5	NA	Er: 8.2 P: 8.2
SOLOIST-WHF (sotagliflozin)	9 mo	S: 69 P: 70	S: 32.6 P: 34.9	S: 100 P: 100	NA	S: 100 P: 100	S: 1816.8 P: 1741	S: 49.2 P: 50.5	S: 122 P: 122	S: 82.1 P: 83.3	S: 15.3 P: 18.2	S: 66.3 P: 62.7
SCORED (sotagliflozin)	16 mo	S: 69 P: 69	S: 44.3 P: 45.5	S: NA P: NA	S: 19.9 P: 20	S: 19.9 P: 19.9	S: 196 P: 198.1	S: 44.4 P: 44.7	S: 138 P: 139	S: 87.5 P: 86.9	S: 1.2 P: 1.2	S: 15.3 P: 14.7

ACEI = angiotensin converting enzyme inhibitors, ARB = angiotensin receptor blocker, ARNI = angiotensin receptor-neprilysin inhibitor, C = canagliflozin, CV = cardiovascular, D = dapagliflozin, DM = diabetes mellitus, E = empagliflozin, eGFR = estimated glomerular filtration rate, Er = ertugliflozin, HF = heart failure, MI = myocardial infarction, MRA = mineralocorticoid receptor antagonist, NA = not applicable (a lack of outcomes reported in the original studies), NT-pro BNP = N- terminal pro-brain natriuretic peptide, P = placebo, S = sotagliflozin, SBP = systolic blood pressure.

* Any RAAS inhibitor including ACEI/ARB, ARNI and MRA.

The DEFINE-HF, DAPA-HF, EMPA-RESPONSE-AHF, EMPEROR-Reduced and SOLOIST-WHF trials focused on HF patients, while the other 5 trials included HF and non-HF patients. The meta-analyses pooled the data from the above 10 studies to compare the total number of CV deaths or HHF between SGLT2 inhibitors versus placebos in HF and non-HF patients.

### 3.3. Risk of bias assessment

The quality assessment results are shown in Figures S2 and S3, Supplementary Digital Content, http://links.lww.com/MD/I238. Except for the DECLARE-TIMI 58 and DEFINE-HF trials, the others had a low risk of bias regarding adequate randomization, allocation concealment and sequence descriptions. All trials did the blinding for the participants, but 6 did not describe the blinding for assessors clearly. Every study had low rates of loss to follow-up, and they all carried out analyses by available intention-to-treat protocols.

### 3.4. Primary outcomes

**Figure 1.**
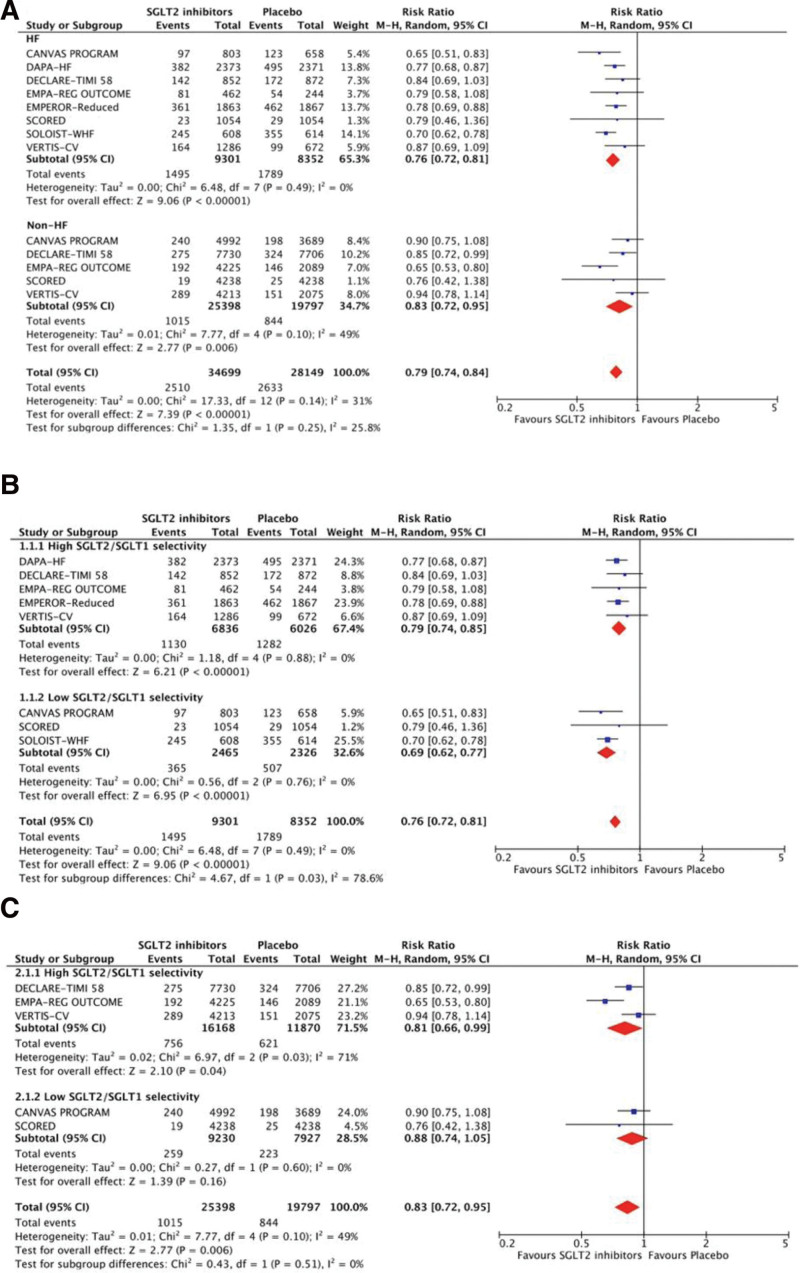
Treatment effects of SGLT2 inhibitors on total cardiovascular death or hospitalization for HF. (A) Treatment effects are stratified by patients with and without HF. (B) Treatment effects are stratified by high and low SGLT2/SGLT1 selectivity in patients with HF. (C) Treatment effects are stratified by high and low SGLT2/SGLT1 selectivity in patients without HF. CI = confidence interval, HF = heart failure, M–H = Mantel–Haenszel, SGLT2 = sodium-glucose co-transporter-2.

The risks of total CV death or HHF were significantly lower in the SGLT2-inhibitor group than the placebo (RR 0.79, 95% CI 0.74–0.84, *P* < .01, *I*^2^ = 31%), and the risk reductions were consistently significant, irrespective of patients with or without HF (Fig. 1). In patients with HF, SGLT2 inhibitors with high and low SGLT2/SGLT1 selectivity led to significantly reduced risks; nevertheless, the low selectivity inhibitors did not have significant risk reduction for patients without HF.

### 3.5. Secondary outcomes

**Figure 2.**
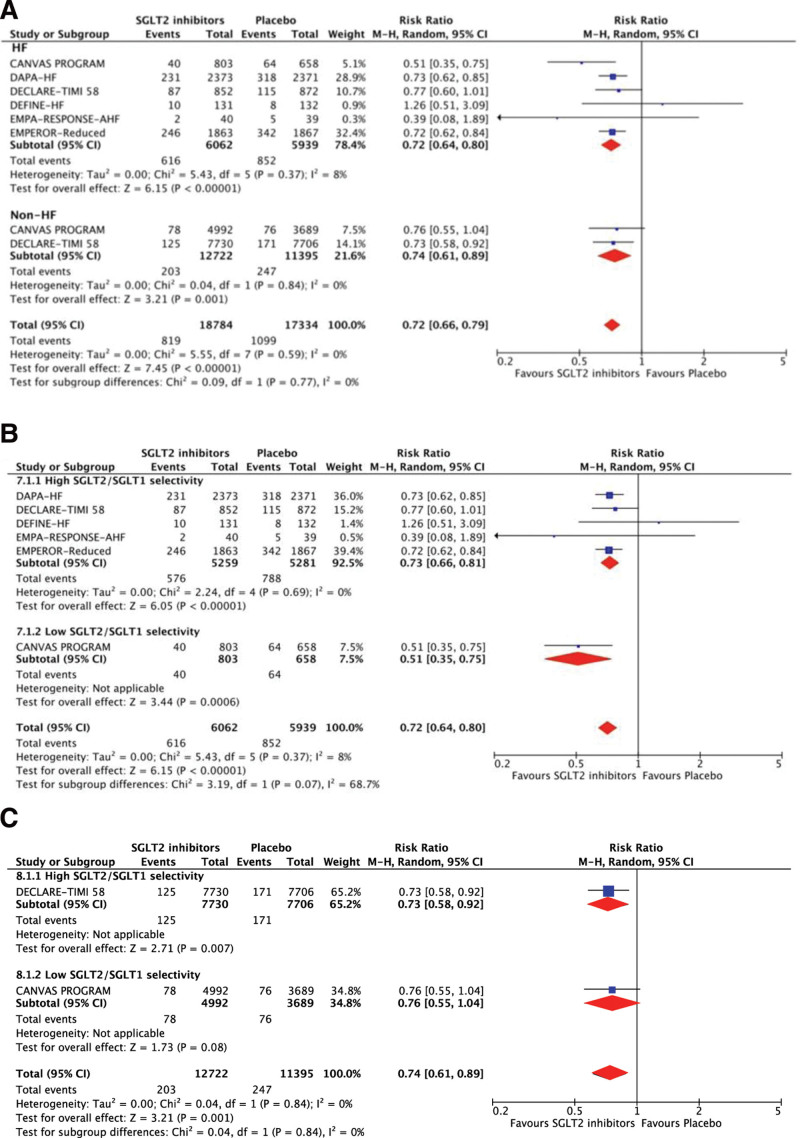
Treatment effects of SGLT2 inhibitors on hospitalization for HF. (A) Treatment effects are stratified by patients with and without HF. (B) Treatment effects are stratified by high and low SGLT2/SGLT1 selectivity in patients with HF. (C) Treatment effects are stratified by high and low SGLT2/SGLT1 selectivity in patients without HF. CI = confidence interval, HF = heart failure, M–H: Mantel–Haenszel, SGLT2 = sodium-glucose co-transporter-2.

The RR of HHF between SGLT2 and placebo was 0.72 (95% CI 0.66–0.79, *P* < .01, *I*^2^ = 0%), and significant risk reductions were noted in both HF and non-HF groups (Fig. 2). SGLT2 inhibitors with high and low selectivity for patients with HF contributed to significantly reduced risks of HHF, but only the high selectivity inhibitors have the effect in patients without HF.

**Figure 3.**
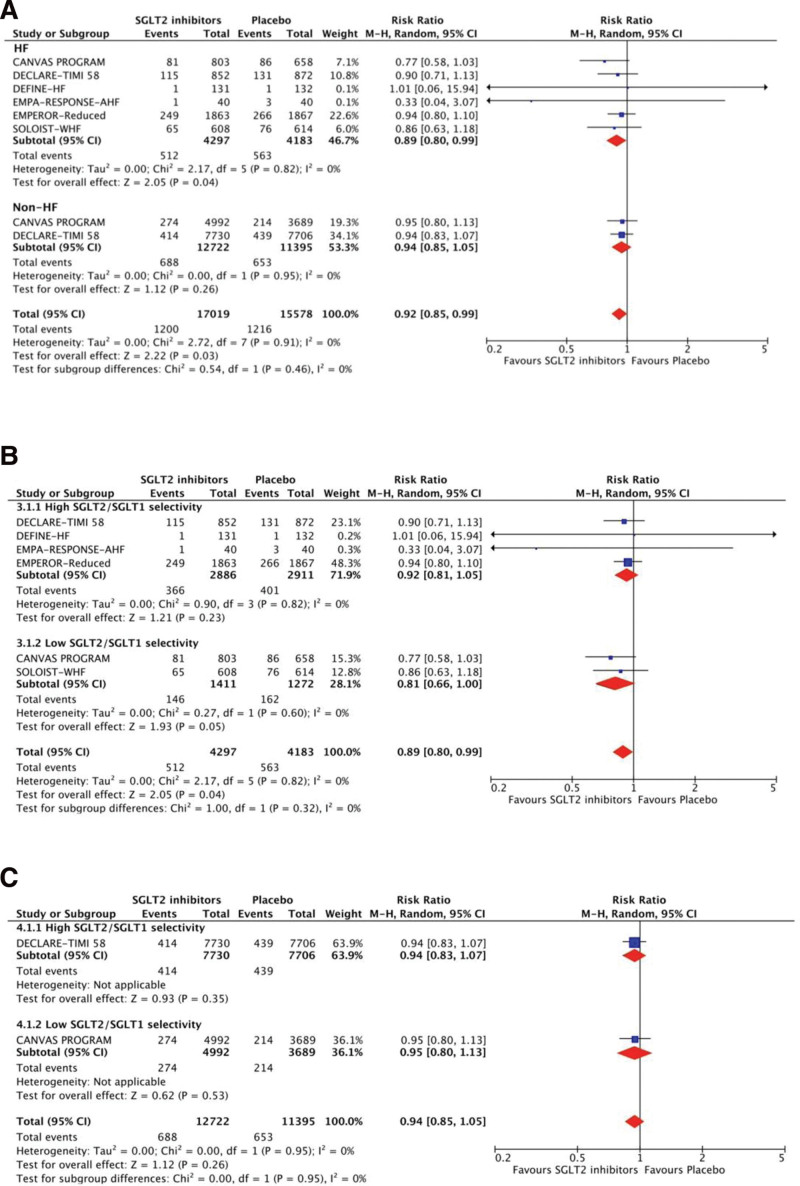
Treatment effects of SGLT2 inhibitors on all-cause mortality. (A) Treatment effects are stratified by patients with and without HF. (B) Treatment effects are stratified by high and low SGLT2/SGLT1 selectivity in patients with HF. (C) Treatment effects are stratified by high and low SGLT2/SGLT1 selectivity in patients without HF. CI = confidence interval, HF = heart failure, M–H = Mantel–Haenszel, SGLT2 = sodium-glucose co-transporter-2.

**Figure 4.**
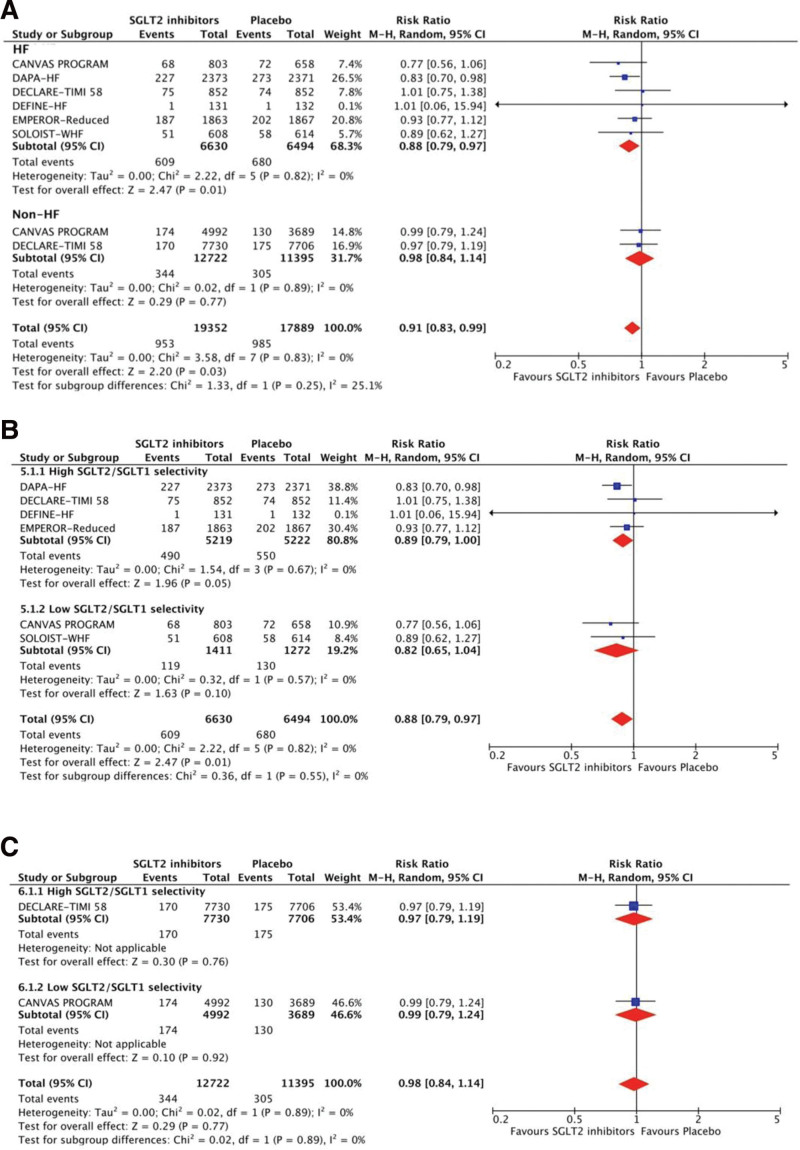
Treatment effects of SGLT2 inhibitors on cardiovascular death. (A) Treatment effects are stratified by patients with and without HF. (B) Treatment effects are stratified by high and low SGLT2/SGLT1 selectivity in patients with HF. (C) Treatment effects are stratified by high and low SGLT2/SGLT1 selectivity in patients without HF. CI = confidence interval, HF = heart failure, M–H: Mantel–Haenszel, SGLT2 = sodium-glucose co-transporter-2.

Figures 3 and 4 show the results of the treatment effects of SGLT2 inhibitors on mortality. SGLT2 inhibitors contributed to a significant risk reduction for all-cause mortality (RR 0.92, 95% CI 0.85–0.99, *P* = .04, *I*^2^ = 0%) and CV death (RR 0.91, 95% CI 0.83–0.99, *P* = .01, *I*^2^ = 25.1%) in patients with HF, but insignificant in those without HF. For patients with HF, using SGLT2 inhibitors with low SGLT2/SGLT1 selectivity had non-statistically lower risks of all-cause mortality than placebo, but the inhibitors with high selectivity did for CV death.

**Figure 5.**
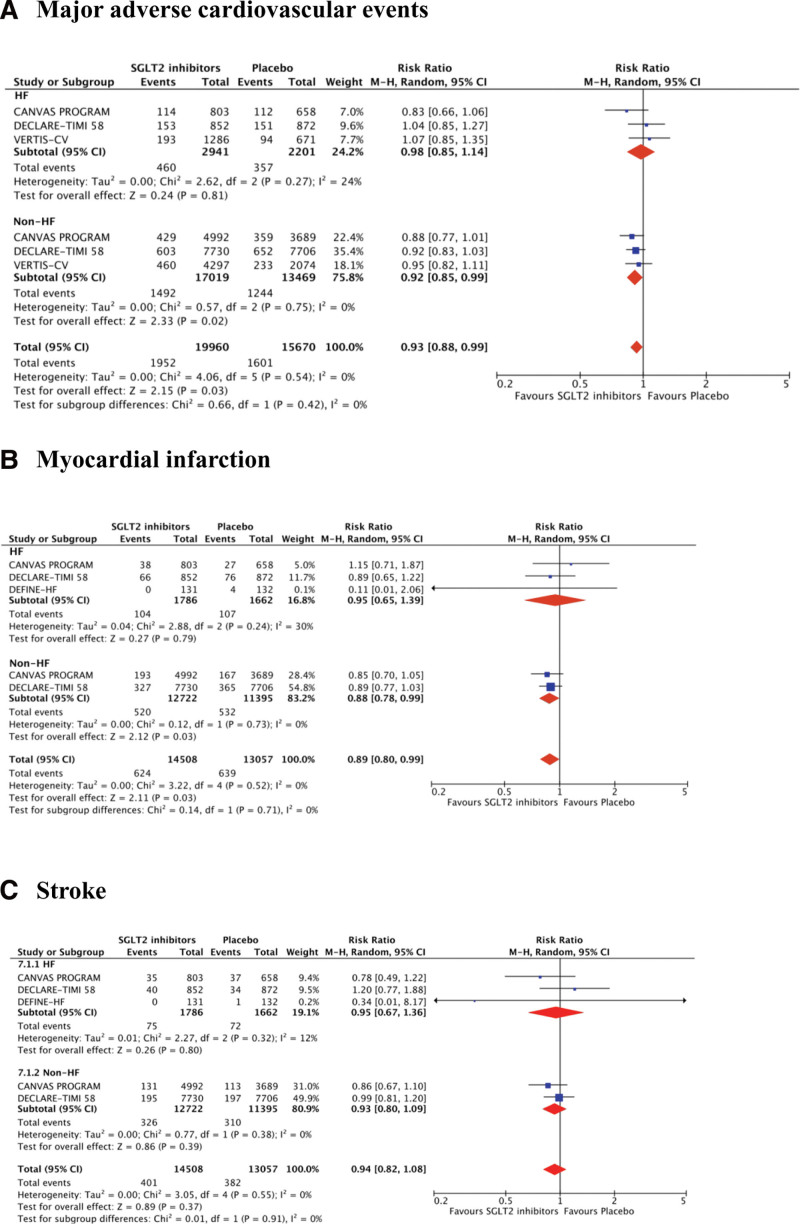
Treatment effects of SGLT2 inhibitors on different clinical cardiovascular outcomes, including (A) major adverse cardiovascular events, (B) myocardial infarction, and (c) stroke. CI = confidence interval, HF = heart failure, M–H = Mantel–Haenszel, SGLT2 = sodium-glucose co-transporter-2.

For the other CV outcomes, SGLT2 inhibitor contributed to risk reductions of MACE (RR 0.92, 95% CI 0.88–0.99, *P* = .03, *I*^2^ = 0%) and myocardial infarction (RR 0.89, 95% CI 0.80–0.99, *P* = .03, *I*^2^ = 0%) in patients without HF. The stroke risks between SGLT2 inhibitors and placebo treatments were not significantly different irrespective of patients with or without HF (Fig. 5).

### 3.6. Subgroup analyses

Canagliflozin, dapagliflozin, empagliflozin and sotagliflozin had a significantly lower risk of total CV death or HHF in HF patients, but in non-HF patients, only dapagliflozin and empagliflozin showed the significantly lower risks. If only considering HHF, canagliflozin, dapagliflozin, and empagliflozin showed a significantly lower risk in HF patients, but of them, only dapagliflozin and canagliflozin did in non-HF patients. All SGLT2 inhibitors did not show a significantly lower risk for CV death, irrespective of HF or non-HF patients (Table S2, Supplementary Digital Content, http://links.lww.com/MD/I239).

## 4. Discussion

This systematic review and meta-analysis, including 52,607 patients, demonstrated the different benefits of SGLT2 inhibitors in CV outcomes, including HHF, CV death, all-cause mortality, MACE and myocardial infarction, except ischemic stroke. Treatment with SGLT2 inhibitors for patients with or without HF contributed to significant risk reductions of total CV death or HHF. Despite the efficacy of HHF in patients with or without HF, SGLT2 inhibitors with high SGLT2/SGLT1 selectivity had more evident treatment effects on reducing HHF in patients without HF. Besides, SGLT2 inhibitors were more advantageous in lowering the risks of MACE and myocardial infarction for patients without HF, but the benefits of mortality reductions were more dominant in HF patients. Among mortality in patients with HF, SGLT2 inhibitors with low SGLT2/SGLT1 selectivity may be beneficial for reducing all-cause mortality, but only inhibitors with high selectivity for reducing CV death.

Previous studies have shown the risk reduction of HHF, CV events and mortality by treating type 2 diabetes with SGLT2 inhibitors.^[6–8,17,30]^ Particularly, the regimen poses more clinical benefits in those with higher CV risks.^[31,32]^ Apart from diabetes, there have been 3 large-scale HF-specific trials on SGLT2 inhibitors (DAPA-HF, EMPA-Reduced, and SOLOIST-WHF trials).^[9–11]^ Despite the heterogeneity across the trials, such as symptom severity, concomitant diabetes, or the SGLT2/SGLT1 selectivity, the effect of SGLT2 inhibitors on the composite outcomes of total CV death or HHF was inspiring in managing HF. Our meta-analysis found a 24% reduction in the composite outcomes, echoing the previous results. Given the analyses of the individual outcomes, SGLT2 inhibitors remained its significant advantages in HHF or CV death.

Despite the benefits, the efficacy of SGLT2 inhibitors in mortality remains debatable. In the HF specific RCTs, only dapagliflozin showed remarkably positive results in both CV and all-cause mortality. The benefits of mortality reduction were not significant in the other two SGLT2 inhibitors. Although our findings regarding risk reduction of mortality were consistent with the previous meta-analysis, which only pooled the DAPA-HF and EMPA-Reduced trials,^[21]^ the advantage of mortality reduction was evident in HF patients, not in non-HF patients. Besides, the individual SGLT2 inhibitors in our subgroup analyses did not show a significant effect in all-cause and CV death regardless of the presence or absence of HF. The interpretation of the clinical benefits in mortality for SGLT2 inhibitors should be cautious, and more clinical trials and real-world evidence are warranted to examine its benefits in reducing mortality.

Regarding the different modulatory sites, SGLT1 commonly uptakes dietary glucose and galactose in the intestine, and SGLT2 reabsorbs the filtered glucose in renal tubular systems.^[14]^ The different SGLT inhibitors with dissimilar structures, selectivity and pharmacokinetics may have varied clinical presentations.^[15]^ Among them, dapagliflozin, empagliflozin and ertugliflozin are classified as inhibitors with high SGLT2/SGLT1 selectivity, while canagliflozin and sotagliflozin are defined as low-selectivity inhibitors.^[26]^ Our subgroup analyses found that the SGLT2 inhibitors with low SGLT2/SGLT1 selectivity had more significant effects on risk reduction of HHF in HF patients than those with high selectivity, but the phenomenon was not observed in non-HF patients. The discrepancy probably results from higher SGLT1 inhibition. The upregulation of SGLT1 in myocardial cells of HF patients had been observed in previous studies, and the modulation of SGLT1 might benefit in reversing cardiac remodeling and improving pumping function in HF patients.^[33,34]^ Although the plausible explanation may help to illustrate the reason for more CV benefits of low SGLT2/SGLT1 selectivity inhibitors in HF patients, more studies are needed to examine the hypothesis.

There are some limitations in this study. First, the heterogeneity across trials may influence the result’s robustness due to lacking detailed individual-level data. For example, the follow-up duration varied in the individual studies, and the duration in specific trials was only around 12 to 13 weeks. This situation may lead to overestimating clinical benefits in our pooled analyses because the period would be too short to capture CV events. However, in our sensitivity analyses, the uncertainty can be neglected due to the trial’s small sample size. Moreover, the random-effect model considering methodological heterogeneity was applied to address this issue. Our primary outcomes with low *I*^2^ values can strengthen the interpretation robustness. Second, only a single trial was analyzed in the subgroup analyses for some SGLT2 inhibitors (canagliflozin, empagliflozin, and ertugliflozin), and sotagliflozin was partially omitted due to lacking complete primary outcomes. Insufficient statistical power may influence the interpretation of clinical efficacy. Further trial post hoc analyses of the SGLT2 inhibitors will be crucial and necessary. Third, although 5 out of the 10 RCTs focused on HF patients, and the DAPA-HF, EMPEROR-Reduced, and SOLOIST-WHF trials had large sample sizes, most patients are HF-reduced ejection fraction patients. The interpretation of clinical benefits in overall HF should be careful, and future studies may need to pool more data to evaluate the effects on HF patients with preserved ejection fraction.

## 5. Conclusions

In conclusion, the treatment effects of SGLT2 inhibitors on total CV death or HHF are consistent with the previous studies, irrespective of patients with or without HF. Notably, treatments with SGLT2 inhibitors significantly reduce mortality in patients with HF, but the benefits are more dominant in preventing MACE and myocardial infarction in patients without HF. Different SGLT2/SGLT1 selectivity in SGLT2 inhibitors may differ in treatment effects in patients with and without HF. More clinical trials are warranted to investigate their differences in clinical efficacy.

## Author contributions

**Conceptualization:** Mei-Chuan Lee, Yi-Ming Hua, Chia-Te Liao.

**Data curation:** Mei-Chuan Lee, Yi-Ming Hua, Chun-Ting Yang.

**Formal analysis:** Fang-Hsiu Kuo, Hsin-Ju Tang.

**Investigation:** Wei-Ting Chang.

**Supervision:** Chia-Te Liao.

**Validation:** Yu-Min Lin, Hung-Yu Chang.

**Visualization:** Yu-Min Lin, Sih-Yao Chen.

**Writing – original draft:** Mei-Chuan Lee, Chia-Te Liao.

**Writing – review & editing:** Han Siong Toh.

## Supplementary Material
