# Supplementary material for: Clinical efficacy of SGLT2 inhibitors with different SGLT1/SGLT2 selectivity in cardiovascular outcomes among patients with and without heart failure: A systematic review and meta-analysis of randomized trials
Source: Medicine (Baltimore). 2022 Dec 23;101(51):e32489. doi: 10.1097/MD.0000000000032489 (PMC9794275; doi:10.1097/MD.0000000000032489)
Supplement: Supplementary file 1 [file medi-101-e32489-s001.pdf]

Search Keywords:

congenital heart disease, heart right ventricle failure, heart failure, sodium glucose cotransporter-2 inhibitor*, sodium glucose cotransporter 2 inhibitor*, sodium-glucose co-transporter-2 inhibitor*, sodium-glucose cotransporter-2 inhibitor*, sodium-glucose cotransporter 2 inhibitor*, sodium glucose cotransporter inhibitor*, sodium-glucose cotransporter inhibitor*, sodium-glucose co-transporter inhibitor*, sodium glucose cotransporter inhibitor*, sodium-glucose cotransporter inhibitor*, SGLT2 inhibitor*, SGLT-2 inhibitor*, dapagliflozin, ertugliflozin, empagliflozin, canagliflozin, ipragliflozin, sotagliflozin and luseogliflozin.
