# Supplementary material for: Clinical efficacy of SGLT2 inhibitors with different SGLT1/SGLT2 selectivity in cardiovascular outcomes among patients with and without heart failure: A systematic review and meta-analysis of randomized trials
Source: Medicine (Baltimore). 2022 Dec 23;101(51):e32489. doi: 10.1097/MD.0000000000032489 (PMC9794275; doi:10.1097/MD.0000000000032489)

Supplementary Figure 1. PRISMA flowchart of literature selection. RCTs, randomized controlled trials

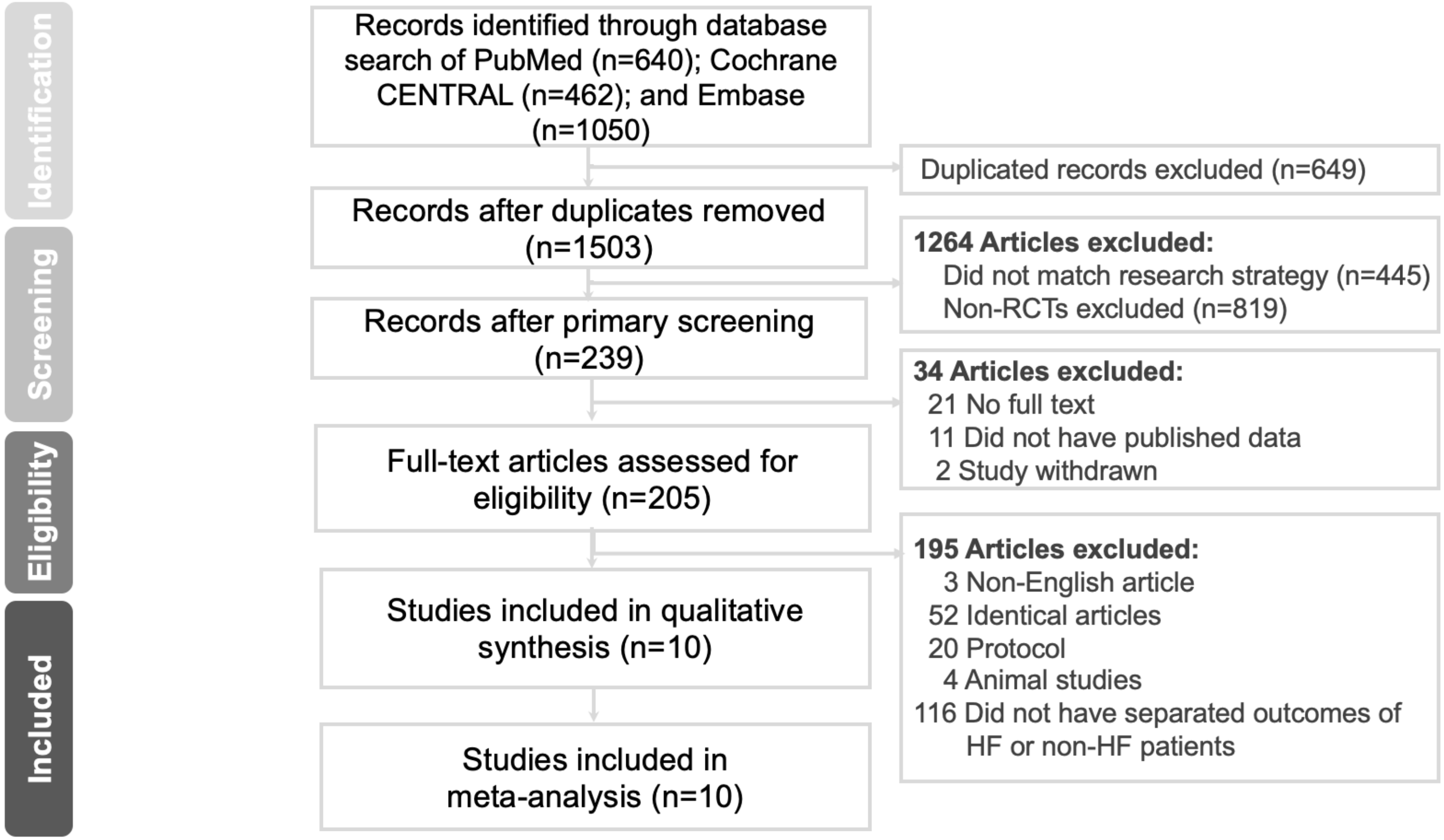

Supplement: Supplementary file 2 [file medi-101-e32489-s002.pdf]
