# Supplementary material for: Clinical efficacy of SGLT2 inhibitors with different SGLT1/SGLT2 selectivity in cardiovascular outcomes among patients with and without heart failure: A systematic review and meta-analysis of randomized trials
Source: Medicine (Baltimore). 2022 Dec 23;101(51):e32489. doi: 10.1097/MD.0000000000032489 (PMC9794275; doi:10.1097/MD.0000000000032489)
Supplement: Supplementary file 3 [file medi-101-e32489-s003.pdf]

Supplementary Table 1. Additional information on the baseline characteristics of the identified studies

Study	Follow up time	Age (mean)	Female (%)	NT-pro BNP (pg/mL)	HF/ LVEF (%)	eGFR mL/min/1.73m2	DM (%) /HbA1c	SBP (mmHg)	MI (%)	ACEI/ ARB (%)	ARNI (%)	MRA (%)
CANVAS PROGRAM (Canagliflozin)	188.2 weeks	C:63.2±8.3<br>P:63.4±8.2	C:35.1<br>P:36.7	NA	C:13.9/NA<br>P:15.1/NA	C:76.7±20.3<br>P:76.2±20.8	C:100/8.2±0.9<br>P:100/8.2±0.9	C:136.4±15.8<br>P: 136.9±15.8	C:55.8<br>P:57.2	RAAS medication *<br>C:80.2<br>P:79.8		
DECLARE-TIMI 58 (Dapagliflozin)	4.2 years	D:63.9±6.8<br>P:64.0±6.8	D:36.9<br>P:27.9	NA	D:9.9/NA<br>P:10.2/NA	D:85.4±15.8<br>P:85.1±16.0	D:100/8.3±1.2<br>P:100/8.3±1.2	D: 135.1±15.3<br>P: 134.8±15.5	D:32.9<br>P:33	D:81.3<br>P:81.3	NA	NA
DEFINE-HF (Dapagliflozin)	13 weeks	D:62.2±11<br>P:60.4±12	D:27.5<br>P:25.8	D:1136<br>P:1136	D:100/27.2±8.0<br>P:100/25.7±8.2	D:66.9±21.1<br>P:71.2±23.1	D:61.8/7.0±1.8<br>P:64.4/7.3±2.0	NA	NA	D:58<br>P:60.6	D:35.9<br>P:28.8	D:58<br>P:63.3

DAPA-HF	18.2	D:66.2±11.0	D:23.8	D:1428	D: 100/31.2±6.7	D: 66.0±19.6	D:41.8/NA	D: 122.0±16.3	NA	D:84.5	D:10.5	D:71.5
(Dapagliflozin)	months	P:66.5±10.8	P:23.0	P:1446	P: 100/30.9±6.9	P: 65.5±19.3	P:41.8/NA	P: 121.6±16.3		P:82.8	P:10.9	P:70.6
EMPA-REG	3.1	E: 63.1 ± 8.6	E:28.8	NA	E:9.9/NA	E: 74.2 ± 21.6	E: 100/8.07 ± 0.85	E: 135.3 ± 16.9	E:46.7	E:81	NA	E:6.5
OUTCOME	years	P: 63.2 ± 8.8	P:28		P:10.5/NA	P: 73.8 ± 21.1	P: 100/8.08 ± 0.84	P: 135.8 ± 17.2	P:46.4	P:80.1		P:5.8
(Empagliflozin)												
EMPA-	12	E:79	E:40	E:4406	E:100/36±17	E:55±18	E:38/NA	E: 127±22	E:30	E:45	E:5	E:48
RESPONSE-	weeks	P:73	P:16	P:6168	P:100/37±14	P:55±18	P:28/NA	P: 121±25	P:38	P:50	P:3	P:45
AHF												
(Empagliflozin)												
EMPEROR-	16	E:67.2±10.8	E:23.5	E:1887	E:100/27.7±6.0	E:61.8±21.7	E:49.8/NA	E: 122.6±15.9	NA	E:70.5	E:18.3	E:70.1
Reduced	months	P:66.5±11.2	P:24.4	P:1926	P:100/27.2±6.1	P:62.2±21.5	P:49.8/NA	P: 121.4±15.4		P:68.9	P:20.7	P:72.6

(Empagliflozin)												
VERTIS-CV	3.5	Er: 64.4±8.1	Er:29.7	NA	Er:23.4/NA	Er: 76.1±20.9	Er: 100/8.2±1.0	Er: 133.5±13.7	Er:47.7	Er:80.9	NA	Er:8.2
(Ertugliflozin)	years	P: 64.4±8.0	P:30.7		P:24.5/NA	P: 75.7±20.8	P: 100/8.2±0.9	P: 133.1±13.9	P:48.4	P:81.5		P:8.2
SOLOIST-WHF	9	S:69	S:32.6	S:1816.8	S:100/35	S:49.2	S:100/7.1	S:122	NA	S:82.1	S:15.3	S:66.3
(Sotagliflozin)	months	P:70	P:34.9	P:1741	P:100/35	P:50.5	P:100/7.2	P:122		P:83.3	P:18.2	P:62.7
SCORED	16	S:69	S:44.3	S:196	S:19.9/NA	S:44.4	S:NA/8.3	S:138	S:19.9	S:87.5	S:1.2	S:15.3
(Sotagliflozin)	months	P:69	P:45.5	P:198.1	P:19.9/NA	P:44.7	P:NA/8.3	P:139	P:20	P:86.9	P:1.2	P:14.7

*Any RAAS inhibitor including ACEI/ARB, ARNI and MRA. NT-pro BNP , N- terminal pro-brain natriuretic peptide; HF, heart failure; LVEF, left ventricular ejection fraction; eGFR, estimated Glomerular filtration rate; DM, diabetes mellitus; SBP, systolic blood pressure; MI, myocardial infarction; ACEI, angiotensin Converting Enzyme Inhibitors; ARB, angiotensin receptor blocker; ARNI, angiotensin receptor-neprilysin inhibitor; MRA, mineralocorticoid receptor antagonist; NA, not applicable (a lack of outcomes reported in the original studies), C, canagliflozin; P, placebo; D, dapagliflozin; E, empagliflozin; Er, ertugliflozin; S, sotagliflozin.
