# Supplementary material for: Clinical efficacy of SGLT2 inhibitors with different SGLT1/SGLT2 selectivity in cardiovascular outcomes among patients with and without heart failure: A systematic review and meta-analysis of randomized trials
Source: Medicine (Baltimore). 2022 Dec 23;101(51):e32489. doi: 10.1097/MD.0000000000032489 (PMC9794275; doi:10.1097/MD.0000000000032489)
Supplement: Supplementary file 5 [file medi-101-e32489-s005.pdf]

Supplementary Table 2. Different treatment effects of the individual SGLT2 inhibitors with different SGLT2/SGLT1 selectivity in the cardiovascular outcomes

	**HF group (study number, RR, 95% CI, I^2)**	**Non-HF group (study number, RR, 95% CI, I^2)**
**Cardiovascular death or hospitalization for heart failure**		
Dapagliflozin vs placebo*	N=2, 0.79 (0.71-0.88, I^2=0%)	N=1, 0.85 (0.72-0.99)
Empagliflozin vs placebo*	N=2, 0.78 (0.70-0.88, I^2=0%)	N=1, 0.65 (0.53-0.80)
Canagliflozin vs placebo*	N=1, 0.65 (0.51-0.83)	N=1, 0.90 (0.75-1.08)
Ertugliflozin vs placebo	N=1, 0.87 (0.69-1.09)	N=1, 0.94 (0.78-1.14)
Sotagliflozin vs placebo	N=2, 0.70 (0.62-0.79, I^2=0%)	N=1, 0.76 (0.42-1.38)
High SGLT2/SGLT1 selectivity*	N=5, 0.79 (0.74-0.85, I^2=0%)	N=3, 0.81 (0.66-0.99, I^2=71%)
Low SGLT2/SGLT1 selectivity	N=3, 0.69 (0.62-0.77, I^2=0%)	N=2, 0.88 (0.74-1.05, I^2=0%)
**Cardiovascular death**		
Dapagliflozin vs placebo	N=3, 0.87 (0.75-1.01, I^2=0%)	N=1, 0.97 (0.79-1.19)
Empagliflozin vs placebo	N=1, 0.93 (0.77-1.12)	NA
Canagliflozin vs placebo	N=1, 0.77 (0.56-1.06)	N=1, 0.99 (0.79-1.24)
Ertugliflozin vs placebo	NA	NA
Sotagliflozin vs placebo	N=1, 0.89 (0.62-1.27)	NA

High SGLT2/SGLT1 selectivity	N=4, 0.89 (0.79-1.00, I^2=0%)	N=1, 0.97 (0.79-1.19)
Low SGLT2/SGLT1 selectivity	N=2, 0.82 (0.65-1.04, I^2=0%)	N=1, 0.99 (0.79-1.24)
**Hospitalization for heart failure**		
Dapagliflozin vs placebo*	N=3, 0.75 (0.65-0.86, I^2=0%)	N=1, 0.73 (0.58-0.92)
Empagliflozin vs placebo	N=2, 0.72 (0.62-0.83, I^2=0%)	NA
Canagliflozin vs placebo	N=1, 0.51 (0.35-0.75)	N=1, 0.76 (0.55-1.04)
Ertugliflozin vs placebo	NA	NA
Sotagliflozin vs placebo	NA	NA
High SGLT2/SGLT1 selectivity*	N=4, 0.73 (0.66-0.81, I^2=0%)	N=1, 0.73 (0.58-0.92)
Low SGLT2/SGLT1 selectivity	N=1, 0.51 (0.35-0.75)	N=1, 0.76 (0.55-1.04)

Given that the groups included more than two studies, the statistical heterogeneity was assessed using I^2 statistics to quantify the proportion of total outcome variability across the studies.

*means statistical significance

HF, heart failure; RR, risk ratio; CI, confidence interval; NA, not applicable (a lack of outcomes reported in the original studies)
